# Long term follow up of patients after allogeneic stem cell transplantation and transfusion of HSV-TK transduced T-cells

**DOI:** 10.3389/fphar.2015.00076

**Published:** 2015-04-23

**Authors:** Eva M. Weissinger, Sylvia Borchers, Anna Silvani, Elena Provasi, Marina Radrizzani, Irene K. Beckmann, Claudia Benati, Joerg Schmidtke, Wolfgang Kuehnau, Patrick Schweier, Susanne Luther, Ivonne Fernandez-Munoz, Gernot Beutel, Fabio Ciceri, Chiara Bonini, Arnold Ganser, Bernd Hertenstein, Michael Stadler

**Affiliations:** ^1^Laboratory for Transplantation Biology, Department of Hematology/Hemostasis/Oncology/Stem Cell Transplantation, Hannover Medical SchoolHannover, Germany; ^2^MolMed, MilanoItaly; ^3^Cancer Immunotherapy and Gene Therapy Program, San Raffaele HospitalMilano, Italy; ^4^Institute of Human Genetics, Hannover Medical SchoolHannover, Germany; ^5^Department of Hematology/Oncology, Klinikum Bremen-MitteBremen, Germany

**Keywords:** gene transfer, horizontal, gene therapy, proteomics data, allogeneic stem cell transplantation, graft vs. host disease

## Abstract

Allogeneic stem cell transplantation (allo-HSCT) is one of the curative treatments for hematologic malignancies, but is hampered by severe complications, such as acute or chronic graft-versus-host-disease (aGvHD; cGvHD) and infections. CD34-selection of stem cells reduces the risk of aGvHD, but also leads to increased infectious complications and relapse. Thus, we studied the safety, efficacy, and feasibility of transfer of gene modified donor T-cells shortly after allo-HSCT in two clinical trials between 2002 and 2007 and here we compare the results to unmodified donor leukocyte infusion (DLI). The aim of these trials was to provide patients with the protection of T-cells after T-cell-depleted allo-HSCT in the matched or mismatched donor setting with an option to delete transduced T-cells, if severe aGvHD occurred within the trial period. Donor-T-cells were transduced with the replication-deficient retrovirus SFCMM-3, expressing HSV-TK and the truncated ΔLNGFR for selection of transduced cells. Transduced cells were transfused either after day +60 (matched donors) or on day +42 (haploidentical donors). Nine patients were included in the first trial (MHH; 2002 until 2007), two were included in TK007 (2005–2009) and six serves as a control group for outcome after haploidentical transplantation without HSV-TK-transduced DLI. Three patients developed acute GvHD, two had grade I of the skin, one had aGvHD on day +131 (post-HSCT; +89 post-HSV-TK DLI) grade II, which was successfully controlled by ganciclovir (GCV). Donor chimerism was stabilized after transfusion of the transduced cells in all patients treated. Functionality of HSV-TK gene expressing T-cells was shown by loss of bcr-able gene expression as well as by control of cytomegalovirus-reactivation. To date, six patients have relapsed and died, two after a second hematopoietic stem cell transplantation without T-cell depletion or administration of unmodified T-cells. Eleven patients (seven post-HSV-TK DLI) are alive and well to date.

## Introduction

Allogeneic hematopoietic stem cell transplantation (allo-HSCT) is applied successfully to the treatment of many hematopoietic malignancies, but remains limited by severe acute graft-versus-host-disease (aGvHD). Despite prophylactic treatment of the patients with immunosuppressive drugs after allo-HSCT, GvHD is still associated with non-relapse mortality (NRM) and contributes around 25% of NRM. (Mavroudis et al., 1998) T-cell depletion of the graft by CD34-enrichment has been found to be the most effective method to prevent GvHD, but this leads to an increased risk of leukemic relapse in about 80% of patients (Horowitz et al., 1990; Gratwohl, 1994). Leukemic relapse can be treated by donor leukocyte infusion (DLI; Hertenstein et al., 1993; Kolb et al., 1995; Massenkeil et al., 2003), providing evidence for an immunological graft-versus-leukemia effect (GvL). Thus, prophylactic transfusion of donor T-cells has been included in many protocols, despite the increased risk for acute GvHD (Kolb, 2008). Acute GvHD contributes significantly to non-relapse morbidity and mortality (NRM), thus prevention or control of this severe complication is necessary.

T-cells are optimal targets for retroviral gene transfer and stability of transduced T-cells has been shown even for several years (Herve et al., 1992; Mavilio et al., 1994; Tiberghien, 1994; Contassot et al., 2000; Weissinger et al., 2000; Ciceri et al., 2009; Borchers et al., 2011). The expression of suicide genes has been shown to be effective to control GvHD, while retaining the positive effects of T-cells like GvL-activity (Bonini et al., 1997; Bondanza et al., 2006; Ciceri et al., 2009; Borchers et al., 2011). Clinical trials with HSV-TK transduced donor T-cells were initiated early in France, Italy and Germany and have been previously published (Bonini et al., 1997; Ciceri et al., 2009; Borchers et al., 2011). Transduced donor T-cells were transfused either immediately after hematopoietic stem cell transplantation (HSCT) on day 0 (Contassot et al., 2000; Ferrand et al., 2000), on day +40 (Ciceri et al., 2009) or after day +60 (Borchers et al., 2011). Eight patients from the first NGFR-HSV-TK-studies developed acute (*n* = 6) or chronic GvHD (*n* = 2), which resolved after treatment with GCV alone in seven of eight patients. Immunization against HSV-TK epitopes was observed in one patient at MHH and led to premature elimination of transduced T cells (Borchers et al., 2011). The chance to get immunized strictly depended on the presence of an active immune system at the time of transfusion of transduced T-cells (Traversari et al., 2007). At Hannover proteomic monitoring was added to predict pending, severe aGvHD to patients included after 2005 [10 of 12 acute myeloid leukemia (AML) patients; Weissinger et al., 2007, 2013]. Here, we analyzed the long term outcome of all patients treated at MHH with genetically modified T-cells and compare the outcome of mismatched transplantation in combination with prophylactic DLI to unmodified DLI-treatment of relapse.

## Materials and Methods

### Study Protocol

#### Case Description

**FIGURE 1:**
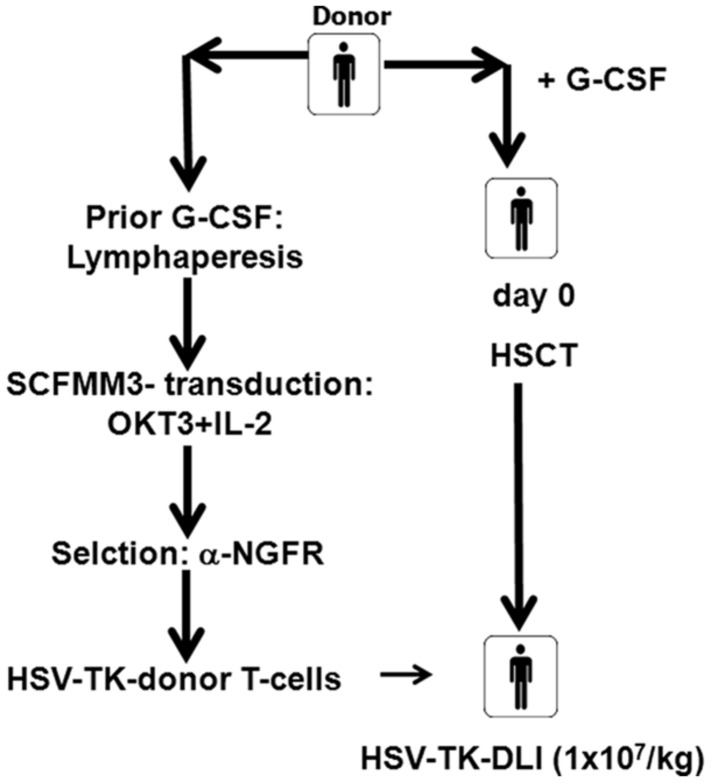
**Clinical trial flow chart.** Prior to G-Lymphapheresis were obtained prior to G-CSF-stimulation of the donor and shipped to MolMed for transduction by courier. After G-CSF-stimulation peripheral blood stem cells (PBSCs) were collected and CD34^+^ cells were selected and transplanted on day 0. The recipient of matched related donor PBSC received conditioning treatment with total body irradiation (TBI; 12 Gy) and cyclophosphamide (120 mg) and ATG (30 mg/kg). The recipients of the mismatched related donor PBSC received conditioning with Fludarabine, Melphalan, Thiotepa, and anti-thymoglobulin (ATG; 60 mg/kg). Lymphapheresis cells were transduced with SCFMM3 , and shipped to Hannover prior transfusion. HSV-TK DLI (1 × 10^7^ cells/kg BW) were administered either after day +60 (MRD) or on day +42 (MMRD; 1 × 10^6^/kg BW).

Seventeen patients, 15 with AML and two with chronic myelogenous leukemia (CML), were transplanted from their HLA-identical (*n* = 9) or haploidentical (*n* = 8) family donors with CD34-enriched stem cells without further immunosuppression (**Table 1**). Eleven received transduced donor lymphocytes according to either one of the protocols (**Figure 1**). The clinical protocols were approved by the ethic committee of the Hannover Medical School (protocol numbers 2157 or 3644) and by the national committee for somatic gene therapy of the “Bundesärztekammer” (No 53 or No 76) and the Paul-Ehrlich-Institute (1274). In addition, both trials were registered at the German register of gene therapy trials.

**Table 1A:** Patient clinical characteristics: all patients were transplanted with CD34-enriched donor cells from their HLA-identical siblings or haploidentical family donors.

Table 1: clinical data	All patients (*n* = 17)	Table 1	All patients (*n* =	17)
**Age**	35 (18–63)	**GvHD-Pro**	
**Disease**		CSA/MTX	0
Acute (AML, ALL, sAML)	15	CSA/MMF	1
Chronic (MDS, MPS, CML, CLL)	2	TCD	11
**Status**		**ATG, Thymo**	14
CR 1/CP1	14	None	3
CR 2 or higher	2	**Donor**	
No CR (untreated, relapse, refr.)	1	Related	17
**Conditioning**		Unrelated	0
Myeloablative (TBI/Cy)	9	**HLA-match**	
RIC	8	Matched	9
**Graft**		Mismatched	8
PBSC	17	**Gender**	
Bone marrow	1^∗^	Female/male	9/8
**HSV-TK DLI**	11	m/f donor	4
None	6	**Alive**	**11**

Donors’ gender and age are shown under “donor.” Transduced T-cells were transfused after day +60 or day +42 (MMRD). ^∗^PBSC (CD6-depleted) +BM.

**Table 1B:** Patient follow up after sibling (MRD) HSCT and HSVTk transfer: acute and chronic GvHD after HSCT or DLI were monitored and are shown in table.

UPN	aGvHD	cGvHD	DLI	aGvHD_DLI (days)	aGvHD_ DLI grade	cGvHD_ DLI	cGvHD_ DLI grade (days)	Days_ HSCT	Relapse	relapse_day	Years HSCT_FU	Survival	comments/cause of death
914	No	No	Yes	Yes	4 (93)	Yes	Mild (451)	451	No		12,4	Yes	
919	No	No	Yes	No		No			No		12,2	Yes	
1021	No	No	Yes	No		Yes	Severe (2626)	2626	Yes	2452	10,6	Yes	ECP for chronic
1040	No	No	Yes	No		No			Yes	960	**5,2**	No	relapse
1048	No	No	Yes	No		No			No		7,6	Yes	
1108	No	No	Yes	No		No			No		9,9	Yes	
1159	No	No	Yes	Yes	1 (192)	No			No		8,1	Yes	
1190	No	No	Yes	No		No			No		8,6	Yes	
1208	No	No	Yes	No		No			Yes	435	**2,0**	No	
**1208**	Yes	No	No	n.a.	3 (97)				No		5,7	No	MOF sepsis post-2nd HSCT
**1040**	No	No	Yes	No		No			Yes	520	2,5	No	Relapse post-2nd HSCT

In addition relapse, survival and cause of death are summarized. Two patients relapsed and have been transplanted for a second time. Outcome of second HSCT shown in table. n.a., not applicable; **FU** (follow up until death after 2nd HSCT); **1040 and 1208,** 2nd HSCT due to relapse.

**Table 1C:** Clinical characteristics, and follow-up after haplo-HSCT (MMRD) with and without HSV-TK gene transfer: patient and donor characteristics acute and chronic GvHD are summarized for two patients with HSV-TK T-cells and six control patients without gene transduced DLI.

UPN	Age	Gender	DIAGN_PR	FAB- classification	Status	HLA-donor	Source	Conditioning	GvHD_Proph	Acute GvHD	aGvHD-days	aGvHD_grade	ChrGvHD	CGvHD grade (days)
1505	23	f	AML	M4/M5	CR 1	MMRD	PBPC	Flu/Mel ph/Thiotepa/ATG	TCD	Yes (post-DLI)	131	II	No	
1550	25	m	AML	MO	Induction failure	MMRD	KM+PBPC	Flamsa(TBI)/ATG	CSA/MTX	No	n.a.		Unknown	
1551	32	f	AML	M5b	CR 2	MMRD	PBPC	Flu/Mel ph/Thiotepa/ATG	TCD	No	n.a.		n.a.	
1438	34	m	AML	M5b	CR 1	MMRD	PBPC	Flu/Mel ph/Thiotepa/ATG	TCD	No	n.a.		No	
1269	18	m	AML	M5a	CR 1	MMRD	PBPC	Flu/Mel ph/Thiotepa/ATG	TCD	ja	54	I	Yes	
1272	38	m	AML	n.d.	CR 1	MMRD	PBPC	Bu/Cy/ATG	CSA/MTX	No	n.a.		Yes	Limited (131)
1455	63	m	AML	M2	CR 2	MMRD	PBPC	Flu/Mel ph/Thiotepa/ATG	TCD	No	n.a.		Yes	Limited (117)
1524	58	f	AML	M**O**	CR 1	MMRD	PBPC	Flamsa (w/o AMSA)(TBI)/ATG	CSA/MMF	ja	30	I	No	

**Table 1D:** CMV-reactivation and immune reconstitution after MMRD-HSCT: summarizes the data after transplantation for 1505 and 1550 with respect to immune reconstitution of CMV-specific CTL.

UPN	Age	Diagnosis	Tx (Tet-table)	HLA recipient	Donor	HLA-donor CMv+	Days post-HSCT	CD3+/μl	CD8+/μl	CD4+/μl	CD8+ count OK? (> =50)	A0201 % CD3+CD8+	A0201/μl	A2402 % der CD3+CD8+	Absolute number A2402/μ l	All CMV+ T-Zellen/μ l	EBV A0201 % der CD3+CD8+	EBV A0201/μl	Comment(s)
1505	23	AML	2.2.2007	A02/A24	MMRD	A02 A24	-50	1301	517	673	Yes	0.22	1	0.05	0	**1**	0.07	0	Prior HSCT
1505	23	AML	2.2.2007	A02/A24	MMRD	A02 A24	28	3	2	1	No	0.00	0	0.00	0	**0**	0.87	0	
1505	23	AML	2.2.2007	A02/A24	MMRD	A02 A24	55	27	5	13	No	0.00	0	0.00	0	**0**	0.42	0	
1505	23	AML	2.2.2007	A02/A24	MMRD	A02 A24	77	3263	2960	251	Yes	0.01	0	0.06	2	**2**	0.01	0	
1505	23	AML	2.2.2007	A02/A24	MMRD	A02 A24	83	3944	3549	315	Yes	0.01	0	0.12	4	**5**	0.22	8	
1505	23	AML	2.2.2007	A02/A24	MMRD	A02 A24	90	2071	1603	386	Yes	0.03	2	2.04	33	**35**	1.81	29	Clear population
1505	23	AML	2.2.2007	A02/A24	MMRD	A02 A24	97	1629	1065	452	Yes	0.02	0	2.75	29	**30**	1.74	19	GVHD II skin
1505	23	AML	2.2.2007	A02/A24	MMRD	A02 A24	137	1199	685	447	Yes	0.01	0	0.02	0	**0**	0.07	0	GVHD II skin
1505	23	AML	2.2.2007	A02/A24	MMRD	A02 A24	143	1048	422	538	Yes	0.02	0	3.01	13	**13**	1.92	8	
1505	23	AML	2.2.2007	A02/A24	MMRD	A02 A24	213	612	391	160	Yes	0.00	0	0.05	0	**0**	0.00	0	
1505	23	AML	2.2.2007	A02/A24	MMRD	A02 A24	230	715	473	205	Yes	0.00	0	0.07	0	**0**	0.00	0	
1550	25	AML	31.5.2007	A02	MMRD	A02	-29	542	232	238	Yes	1.70	4	n.a.	n.a.	**4**	0.12	0	Prior HSCT
1550	25	AML	31.5.2007	A02	MMRD	A02	32	23	16	2	No	1.06	0	n.a.	n.a.	**0**	0.00	0	
1550	25	AML	31.5.2007	A02	MMRD	A02	43	630	434	62	Yes	0.13	1	n.a.	n.a.	**1**	0.00	0	

Prior HSCT (day -29) CMV-CTL could be detected, after HSCT the patient reactivated CMV und by day +32 CMV-CTL reconstitution occurred. Shown are absolute numbers of CMV-CTL after tetramer staining with HLA-^∗^A02 (pp65-NLV) tetramer. Patient 1550 (PBSC-CD6-depleted+ BM) reactivated CMV on day +32 and controlled the CMV-reactivation after treatment with GCV (no transduced DLI) and upon expansion of CMV-CTL from the graft.

### Conditioning and Hematopoietic Stem Cell Transplantation

T-cells were harvested from all donors after informed consent and shipped to Milano for transduction. Eleven patients received total body irradiation (TBI; 12 Gy) and cyclophosphamide (120 mg/kg) followed by a CD34-enriched stem cell graft from matched related donors (MRDs; **Table 1**). Donors received G-CSF (2 × 5 μg/kg daily) for 4–5 days and blood leukocytes were collected at the Institute for Transfusion Medicine (MHH; Borchers et al., 2011). CD34-selection was performed under GMP conditions using the CliniMACS-system (Miltenyi; Bergisch Gladbach, Germany) at the Center for Cellular Therapy (former Cytonet, Hannover, Germany). At least 3.9 × 10^6^/kg CD34-positive cells were transplanted on day 0, and CD3^+^ T-cells were usually below 1 × 10^4^ cells/kg body weight (**Table 1**). CD34-selection was the only GvHD prophylaxis. Missing informed consent, acute GvHD grade II or more, life threatening infections, or relapse at the time of transfusion were exclusion criteria for gene therapy. Relapse and declining donor chimerism were treated with additional, non-transduced DLI (see **Table 2**). One patient (UPN 1505) received a second transduced DLI to treat relapse.

**Table 2:** Summary of transduced and non-transduced DLI: peripheral blood of all patients was screened routinely for the presence of transduced cells with FACS and PCR.

UPN	Gebdat	Age_HSCT (1st and 2nd)	DLI	DLI-days-post-HSCT	Cells/kg BW
914	06.07.1957	45	DLI(HSV-TK)	71	1 × 10E7
914	06.07.1957	45	DLI chimerism	178	1 × 10E7
91*A*	06.07.1957	45	DLI chimerism	249	1 × 10E7
91*A*	06.07.1957	45	DLI chimerism	329	3 × 10E7
91*A*	06.07.1957	45	DLI chimerism	406	5,3 × 10E7
919	03.02.1967	35	DLI(HSV-TK)	87	4 × 10E6
1021	02.12.1963	39	DLI(HSV-TK)	113	1,3 x10E7
1021	02.12.1963	39	DLIrelapse	2535	1,8 × 10E7
1021	02.12.1963	39	DLIrelapse	2564	1,12 × 10E8
1021	02.12.1963	39	DLIrelapse	2592	2 × 10E8
1040	28.12.1952	50	Stemcell boost	191	6 × 10E6 CD34^+^
1040	28.12.1952	50	DLI(HSV-TK)	330	1 × 10E7
1040	28.12.1952	53	DLIprophylaxis	268	1,03 × 10E6
1040	28.12.1952	53	DLIprophylaxis	304	5 × 10E6
1040	28.12.1952	53	DLIprophylaxis	339	1 × 10E7
1040	28.12.1952	53	DLIrelapse	535	5 × 10E7
1040	28.12.1952	53	DLIrelapse	563	1 × 10E8
1040	28.12.1952	53	DLIrelapse	612	1,23 × 10E8
1048	23.01.1965	38	DLI(HSV-TK)	100	1,5 × 10E7
1108	18.02.1953	51	DLI(HSV-TK)	126	4 × 10E6
1159	23.12.1968	35	DLI(HSV-TK)	136	7,5 x10E6
1190	10.03.1966	38	DLI(HSV-TK)	129	1 × 10E7
1208	06.12.1953	51	DLI(HSV-TK)	73	1,3 × 10E7
1208	06.12.1953	51	DLIrelapse	519	1,8 × 10E7
1269	10.04.1987	18	DLIrelapse	377	5,38 × 10E4
1269	10.04.1987	18	DLIrelapse	405	1,3 × 10E5
1269	10.04.1987	18	DLIrelapse	433	4,7 × 10E5
1269	10.04.1987	18	DLIrelapse	461	1,27 × 10E6
1269	10.04.1987	18	DLIrelapse	488	5 × 10E6
1269	10.04.1987	18	DLIrelapse	517	1 × 10E7
1269	10.04.1987	18	DLIrelapse	545	5,03 × 10E7
1438	13.10.1972	34	No	na	
1550	25.04.1982	25	No		
1505	16.12.1983	23	DLI(HSV-TK)	48	1 × 10E6
1505	16.12.1983	23	DLI(HSV-TK) therapy	222	1 × 10E6
1551	18.08.1975	32	DLI(HSV-TK)	42	1 × 10E6

The number of transduced (first DLI) and non-transdcued DLI (for loss of chimerism or relapse) are shown as number of transduced cells (CD3^+^ cells) or as number of leukocytes transfused per kg body weight.

### Description of the Retroviral Vector and the Transduction Protocol

Lymphapheresis material was obtained from 11/17 donors and shipped to MolMed (Italy) by courier for transduction. The replication-deficient, retroviral vector SFCMM-3 encodes the HSV-TK gene that confers sensitivity to GCV, and the truncated low affinity nerve-growth-factor-receptor gene (ΔLNGFR) serving as a positive selection marker and transduction protocols have been described previously (Mavilio et al., 1994; Bonini et al., 2007; Ciceri et al., 2007, 2009; Borchers et al., 2011) Briefly, cells were expanded with OKT-3 (30 ng/ml) in RPMI1640 with 5% autologous plasma and 100 U/ml IL-2 (Chiron, USA) for 72 h. Cells were transduced by spin-inoculation with SFCMM-3-supernatant twice within 24 h in the presence of 4 μg/ml protamine sulfate and expanded for 48 h. Transduction efficiencies were determined by FACS and subsequently the cells were selected using a monoclonal antibody (anti-LNGFR-antibody, Roche, Mannheim, Germany) and immunomagnetic beads (Dynabeads; Verzeletti et al., 1998). Transduced and selected cells were cryopreserved, while safety tests (as approved by the authorities) were accomplished and shipped to Hannover for transfusion (**Table 1**).

### Monitoring for the Presence of Transduced T Lymphocytes

Characterization and *ex vivo* detection of circulating transduced cells was planned at weekly for the first month 1, 2, 3, 4, 8, 12, 16, 20, 24, at 9 months, 12 months, and yearly thereafter. The follow up for three patients is now more than 12 years (**Tables 2** and **3**). Flow cytometry (FACS; Coulter, Germany) was performed to examine the frequency and phenotype of the transferred gene-modified T-cells *in vivo* using mAbs specific to LNGFR (Roche, Mannheim, Germany), CD3, CD4, and CD8 (Coulter), respectively. Immune reconstitution was analyzed for B-, T-, natural killer cells, macrophages, and monocytes.

**Table 3:** Long term follow up of PCR for TK-gene: summarizes the results obtained with PCR on HSV-TK gene expression.

UPN	Days post-HSCT	Days post-DLI	Years follow-up	Material for cDNA	HSV-TK PCR result	Comments
914	1155	1084	3,0	PBMNCs	Positive	
914	4161	4090	11,2	CD3+	Negative	
914	4526	4455	12,4	CD3+	Negative	Alive
919	3664	3577	9,8	CD3+	Negative	
919	4165	4078	11,2	CD3+	Negative	
919	4530	4443	12,2	CD3+	Negative	Alive
1021	857	744	2,0	PBMNCs	Positive	
1021	2831	2718	7,4	CD3+	Negative	
1021	3868	3755	10,60	CD3+	Negative	Alive
1040	687	357	1,0	CD3+	Positive	
1040	974	644	1,8	PBMNCs	Positive	
1040	1057	727	2,90	CD3+	Negative	Relapse; re-transplantation; died
1048	791	691	1,9	PBMNCs	Positive	
1048	1002	902	2,5	PBMNCs	Negative	
1048	2787	2687	7,64	CD3+	Negative	Alive
1108	723	597	1,6	PBMNCs	Positive	
1108	807	681	1,9	PBMNCs	Positive	
1108	3598	3472	9,5	CD3+	Negative	Alive
1159	365	229	0,6	PBMNCs	Negative	
1159	640	504	1,4	CD3+	Negative	
1159	3382	3246	8,9	CD3+	Negative	Alive
1190	2398	2269	6,2	CD3+	Negative	
1190	2767	2638	7,2	CD3+	Positive	
1190	3144	3015	8,3	CD3+	Negative	Late relapse, alive
1208	476	403	1,1	PBMNCs	Positive	
1208	484	411	1,1	PBMNCs	Positive	
1208	512	439	1,2	PBMNCs	Positive	Relapse; re-transplantation; died
1505	221	173	0,5	CD3+	Positive	
1505	250	202	0,6	CD3+	Positive	
1505	277	229	0,6	CD3+	Negative	Died relapse
1551	114	72	0,2	CD3+	Positive	
1551	148	106	0,3	CD3+	Negative	
1551	220	178	0,5	CD3+	Positive	**Died**

UPN# (unique patient identification number), followed by sample date and days after allogeneic HSCT, days after DLI, Tx date (date of transplantation), Days post 1st or 2nd DLI (where appropriate), material from which RNA or DNA were obtained (PBMC: peripheral blood mononuclear cells; CD3: CD3-positive T-cells) and results of HSV-TK-PCR. DNA-isolation and HSV-TK-PCR were done as described in Section “Materials and Methods.”

The presence of gene modified T-cells was confirmed by polymerase chain reactions (PCRs) with primers for the HSV-TK and the LNGFR-gene, as described (Borchers et al., 2011).

### HSV-TK-Gene, Donor-Chimerism, and T-Cell Receptor (TCR)-Vβ Family Expression

Transduced cells were analyzed at above mentioned time points and yearly thereafter for the presence of HSV-TK gene expression. To increase the likelihood to detect gene modified cells, PBMNC were enriched for CD3^+^/CD4^+^ and CD8^+^ T-cells. Genomic DNA was isolated using the QIAamp DNA MinElute Kit (Qiagen, Hilden) and amplified. A nested PCR had been developed for the detection of HSV-TK gene (Borchers et al., 2011). The PCR was run on 1.2% agarose gels and analyzed. A summary of the results is shown in **Table 3**.

### RT-PCR-Analyses

RNA was isolated from PBMNC or CD3-enriched, CD3^+^/CD8^+^ and CD3^+^/CD4^+^ T-cells transcribed into cDNA. TCR-repertoire analyses were done using 25 vß- family specific primers, including controls for the constant region of the TCR and human ß-actin, as described (Naumov et al., 1995). RT-PCR for detection of the *bcr-abl* fusion transcript was performed as proposed by the BIOMED-1 nested PCR on Taqman concerted action (Van Dongen et al., 1999; Borchers et al., 2011). PCR was performed with the T3 thermocycler (Biometra). Donor chimerism was analyzed by PCR amplification of highly polymorphic short tandem repeat (PCR-STR) sequences in peripheral blood and/or bone marrow samples as described earlier (Briones and Amils, 1998).

## Results

### 12 Years of Successful Transduced T-Cell Transfer at MHH

Seventeen patients were transplanted from MRD or mismatched related donors (MMRDs) and eleven received gene-modified donor T-cells on day +42 (*n* = 2) or after day +60 (*n* = 9) after HSCT. Clinical and demographic data are summarized in **Table 1**. Lymphaphereses were prepared from 11 donors and shipped to MolMed for transduction with SCFMM-3 and enrichment for transduced T-cells with magnetic immunobeads yielded purities of more than 90% for ΔLNGFR^+^ cells, ranging from 91 to 99.1%. The patients were scheduled to receive transduced T-cells on day +42 or day +60, respectively, after matched (MRD) or haploidentical (MMRD) HSCT, if the clinical situation of the patients allowed transfusion. The first nine patients (MRD; **Table 1B**), received the T-cell transfusion by day +107 after HSCT (range: 71–330), the two patients receiving cells from MMRD received the HSV-TK DLI on day +42 (**Table 1C**). The immune reconstitution for lymphocytes was 640 CD3^+^ T-cells/μl (range: 150–1000 cells/μl) after MRD HSCT and zero for the MMRD transplant recipients.

### Transfusion of Genetically Modified Donor Lymphocytes or Untransduced

Seven patients had residual host cells prior to DLI. In six patients donor chimerism progressively increased after DLI and full donor chimerism was obtained in three patients, thus suggesting that the infusion of genetically modified donor lymphocytes may have facilitate HSCT engraftment. In T-depleted transplantation the achievement of full chimerism is particularly difficult. In accordance with this hypothesis, expansion of transduced donor lymphocytes often preceded the improvement of donor chimerism.

ΔLNGFR-positive cells were detected by FACS (up to 6.6% of CD3^+^ cells) for at least 6 months after transfusion. In two MMRD-recipients all CD3^+^ T-cells detected were transduced and of donor-origin, as expected. Patient UPN1505 reactivated CMV early after MMRD-HSCT. Transduced cells were transfused on day +42 from the CMV-seropositive donor and by day +77 CMV-CTL (*n* = 2) from the MMRD were detected (**Table 1D**). This was followed by an expansion of the CMV-CTL and led to the control of CMV-reactivation (**Table 1D**). UPN 1505 also developed aGvHD grade II (day +131; **Tables 1C,D**). GCV was given at 5 mg/kg body weight and as a result aGvHD was cleared. Unfortunately the patient developed relapse and died despite a second transfusion of HSV-TK transduced DLI on day +222 (**Tables 1C** and **2**). Patient 1550 did not receive any DLI, but CMV-CTL could be detected by day +44, providing protection against CMV-reactivation. HSV-TK gene expression was analyzed until last follow-up in all patients and a summary of the data is shown **Table 3**. The longest follow-up for HSV-TK transduced cells was 7 years after HSCT (UPN1190), followed by 3 years (UPN914), whereas HSV-TK transduced cells were detectable for almost 2 years in another 6. One patient UPN919 lost HSV-TK gene expression after 22 months, in the absence of GCV treatment due to an immunization against transgene products (Borchers et al., 2011). Thus, seven patients had detectable levels of HSV-TK for more than 1 year after HSCT, two died prior to the first year after HSCT (**Table 3**). PCR for HSV-TK gene expression and donor chimerism are done yearly for all patients alive. To date, six patients have a follow up of more than 6 years patients, two patients had been treated 12 years ago and are both alive and well. None of the surviving patients show HSV-TK expression at last follow up (**Table 3**).

## Discussion

Our data show the long term safety and efficacy of retroviral gene transfer in mature T-cells. The transfusion of transduced cells was tolerated well in all patients with no reported toxicities. Replication competent particles have not been detected and in general gene expression is lost after about 2 years. Ciceri et al. (2009) have recently shown that transfusion of transduced CD3^+^ cells after haploidentical transplantation allows for optimal expansion of transduced cells (Garin et al., 2001). Long term transgene expression for more than 1 year was observed in seven of nine patients, five had HSV-TK expressing cells for more than 1 year. Two patients transplanted from mismatched donors died prior to day +365. Efficacy of the cells had been demonstrated by control of *bcr-abl* positivity, donor-chimerism conversion and in the MMRD-transplanted patient 1505 a cytomegalovirus-reactivation was controlled by the expansion of HSV-TK-transduced donor T-cells.

TK007 protocol called for haploidentical (MMRD) HSCT and eight patients were transplanted from haploidentical donors (MMRD) at Hannover. Only two patients received transduced DLI, the others were observed as control group for MMRD-HSCT and HSV-TK transfer. All patients receiving transduced T-cells expressed the transgenes for at least 1 year or until last follow up, if patients died prior to day +365 (**Table 3**). In one patient an immunization against the transgenes had occurred thus the cells were lost earlier (Borchers et al., 2011).

We have not detected non-functional HSV-TK genes in pour small patient cohort to date (Mercier-Letondal et al., 2008). ΔLNGFR expression as a selection marker allows rapid selection of the transduced cells, which may be favorable for the phenotype of the transduced cells, leaving the T-cell receptor repertoire of the transduced cells relatively intact (Borchers et al., 2011) as compared to more time consuming selection methods with antibiotic resistance genes.

In our studies, two of the 11 patients treated with HSV-TK transduced T-cells so far developed aGvHD grades I or II, respectively. The patient (UPN1505) had developed acute GvHD grade II and was treated successfully with GCV, thus giving a confirmation of the feasibility of the HSV-TK gene transfer for control of acute GvHD by GCV treatment. The persistence of the transduced cells over about 2 years in the majority of the patients suggests that the transduced cells were able to engraft and expand. The expansion of the transduced cells could be linked to clinical data, such as viral infections or was seen as response to declining donor chimerism, suggesting function of transduced cells. After transfusion of the transduced cells one patient reactivated CMV early after HSCT (day +20) and upon transfusion of the transduced cells the CMV-reactivation was cleared. In one patient (UPN919) HSV-TK transduced were lost after 22 months. A low rate of immune mediated elimination of transduced cells was observed in our studies, suggesting that early add-back of TK-expressing cells is permitted, despite intrinsic immunogenicity of the viral-derived suicide gene.

In summary, the data presented here indicate that the use of SFCMM-3 transduced T-cells can be monitored in a clinical setting and is currently safe, efficient, and adequate for the proposed treatment of GvHD.

## Conflict of Interest Statement

MolMed is a industrial partner, some of the authors are associated with MolMed (as indicated by affiliation).

## References

[B1] Bondanza A. Valtolina V. Magnani Z. Ponzoni M. Fleischhauer K. Bonyhadi M. (2006). Suicide gene therapy of graft-versus-host disease induced by central memory human T lymphocytes. *Blood* 107 1828–1836 10.1182/blood-2005-09-3716 16293601

[B2] Bonini C. Bondanza A. Perna S. K. Kaneko S. Traversari C. Ciceri F. (2007). The suicide gene therapy challenge: how to improve a successful gene therapy approach. *Mol. Ther.* 7 1248–1252 10.1038/sj.mt.6300190 17505474

[B3] Bonini C. Ferrari G. Verzeletti S. Servida P. Zappone E. Ruggieri L. (1997). HSV-TK gene transfer into donor lymphocytes for control of allogeneic graft-versus-leukemia. *Science* 276 1719–1724 10.1126/science.276.5319.1719 9180086

[B4] Borchers S. Provasi E. Silvani A. Radrizzani M. Benati C. Dammann E. (2011). Genetically modified donor leukocyte transfusion and graft-versus-leukemia effect after allogeneic stem cell transplantation. *Hum. Gene. Ther.* 7 829–841 10.1089/hum.2010.162 21091264 PMC3135250

[B5] Briones C. Amils R. (1998). The evolution of function: a new method to assess the phylogenetic value of ribosomal sensitivity to antibiotics. *Int. Microbiol.* 4 301–306. 10943378

[B6] Ciceri F. Bonini C. Marktel S. Zappone E. Servida P. Bernardi M. (2007). Antitumor effects of HSV-TK-engineered donor lymphocytes after allogeneic stem-cell transplantation. *Blood* 109 4698–4707 10.1182/blood-2006-05-023416 17327416

[B7] Ciceri F. Bonini C. Stanghellini M. T. Bondanza A. Traversari C. Salomoni M. (2009). Infusion of suicide-gene-engineered donor lymphocytes after family haploidentical haemopoietic stem-cell transplantation for leukaemia (the TK007 trial): a non-randomised phase I-II study. *Lancet. Oncol.* 5 489–500 10.1016/S1470-2045(09)70074-9 19345145

[B8] Contassot E. Ferrand C. Angonin R. Cohen J. L. de Carvalho B. M. Lorchel F. (2000). Ganciclovir-sensitive acute graft-versus-host disease in mice receiving herpes simplex virus-thymidine kinase-expressing donor T cells in a bone marrow transplantation setting. *Transplantation* 69 503–508 10.1097/00007890-200002270-00007 10708102

[B9] Ferrand C. Robinet E. Contassot E. Certoux J. M. Lim A. Herve P. (2000). Retrovirus-mediated gene transfer in primary T lymphocytes: influence of the transduction/selection process and of ex vivo expansion on the T cell receptor beta chain hypervariable region repertoire. *Hum. Gene. Ther.* 11 1151–1164 10.1089/10430340050015202 10834617

[B10] Garin M. I. Garrett E. Tiberghien P. Apperley J. F. Chalmers D. Melo J. V. (2001). Molecular mechanism for ganciclovir resistance in human T lymphocytes transduced with retroviral vectors carrying the herpes simplex virus thymidine kinase gene. *Blood* 97 122–129 10.1182/blood.V97.1.122 11133751

[B11] Gratwohl A. (1994). Bone marrow transplantation today. *Support Care Cancer* 2 27–34 10.1007/BF00355237 8156255

[B12] Hertenstein B. Wiesneth M. Novotny J. Bunjes D. Stefanic M. Heinze B. (1993). Interferon-alpha and donor buffy coat transfusions for treatment of relapsed chronic myeloid leukemia after allogeneic bone marrow transplantation. *Transplantation* 56 1114–1118 10.1097/00007890-199311000-00013 8249110

[B13] Herve P. Flesch M. Tiberghien P. Wijdenes J. Racadot E. Bordigoni P. (1992). Phase I-II trial of a monoclonal anti-tumor necrosis factor alpha antibody for the treatment of refractory severe acute graft-versus-host disease. *Blood* 79 3362–3368. 1596576

[B14] Horowitz M. M. Gale R. P. Sondel P. M. Goldman J. M. Kersey J. Kolb H. J. (1990). Graft-versus-leukemia reactions after bone marrow transplantation. *Blood* 75 555–562. 2297567

[B15] Kolb H. J. (2008). Graft-versus-leukemia effects of transplantation and donor lymphocytes. *Blood* 112 4371–4383 10.1182/blood-2008-03-077974 19029455

[B16] Kolb H. J. Schattenberg A. Goldman J. M. Hertenstein B. Jacobsen N. Arcese W. (1995). Graft-versus-leukemia effect of donor lymphocyte transfusions in marrow grafted patients. *Blood* 86 2041–2450. 7655033

[B17] Massenkeil G. Nagy M. Lawang M. Rosen O. Genvresse I. Geserick G. (2003). Reduced intensity conditioning and prophylactic DLI can cure patients with high-risk acute leukaemias if complete donor chimerism can be achieved. *Bone Marrow Trans.* 31 339–345 10.1038/sj.bmt.1703859 12634724

[B18] Mavilio F. Ferrari G. Rossini S. Nobili N. Bonini C. Casorati G. (1994). Peripheral blood lymphocytes as target cells of retroviral vector-mediated gene transfer. *Blood* 83 1988–1997. 8142665

[B19] Mavroudis D. A. Read E. J. Molldrem J. Raptis A. Plante M. Carter C. S. (1998). T cell-depleted granulocyte colony-stimulating factor (G-CSF) modified allogenic bone marrow transplantation for hematological malignancy improves graft CD34+ cell content but is associated with delayed pancytopenia. *Bone Marrow Trans.* 21 431–440 10.1038/sj.bmt.1701120 9535034

[B20] Mercier-Letondal P. Deschamps M. Sauce D. Certoux J. M. Milpied N. Lioure B. (2008). Early immune response against retrovirally transduced herpes simplex virus thymidine kinase-expressing gene-modified T cells coinfused with a T cell-depleted marrow graft: an altered immune response? *Hum. Gene. Ther.* 9 937–950 10.1089/hum.2007.156 18810797

[B21] Naumov G. I. Naumova E. S. Louis E. J. (1995). Genetic mapping of the alpha-galactosidase MEL gene family on right and left telomeres of *Saccharomyces cerevisiae*. *Yeast* 11 481–483 10.1002/yea.320110512 7597853

[B22] Tiberghien P. (1994). Use of suicide genes in gene therapy. *J. Leukoc. Biol.* 56 203–209. 8071596 10.1002/jlb.56.2.203

[B23] Traversari C. Marktel S. Magnani Z. Mangia P. Russo V. Ciceri F. (2007). The potential immunogenicity of the TK suicide gene does not prevent full clinical benefit associated with the use of TK-transduced donor lymphocytes in HSCT for hematologic malignancies. *Blood* 109 4708–4715 10.1182/blood-2006-04-015230 17327417

[B24] Van Dongen H. P. Olofsen E. VanHartevelt J. H. Kruyt E. W. (1999). A procedure of multiple period searching in unequally spaced time-series with the Lomb-Scargle method. *Biol. Rhythm. Res.* 2 149–177 10.1076/brhm.30.2.149.1424 11708361

[B25] Verzeletti S. Bonini C. Marktel S. Nobili N. Ciceri F. Traversari C. (1998). Herpes simplex virus thymidine kinase gene transfer for controlled graft-versus-host disease and graft-versus-leukemia: clinical follow-up and improved new vectors. *Hum. Gene. Ther.* 9 2243–2251 10.1089/hum.1998.9.15-2243 9794208

[B26] Weissinger E. M. Franz M. Voss C. Bonini C. Kremmer E. Kolb H. J. (2000). Expression of HSV-TK suicide gene in primary T lymphocytes: the dog as a preclinical model. *Cytokines Cell. Mol. Ther.* 1 25–33 10.1080/13684730050515886 10976536

[B27] Weissinger E. M. Metzger J. Dobbelstein C. Wolff D. Schleuning M. Kuzmina Z. (2013). Proteomic peptide profiling for preemptive diagnosis of acute graft-versus-host disease after allogeneic stem cell transplantation. *Leukemia* 28 842–852 10.1038/leu.2013.210 23842427 PMC7101954

[B28] Weissinger E. M. Schiffer E. Hertenstein B. Ferrara J. L. Holler E. Stadler M. (2007). Proteomic patterns predict acute graft-versus-host disease after allogeneic hematopoietic stem cell transplantation. *Blood* 109 5511–5519 10.1182/blood-2007-01-069757 17339419

